# FAEE exerts a protective effect against osteoporosis by regulating the MAPK signalling pathway

**DOI:** 10.1080/13880209.2022.2039216

**Published:** 2022-02-18

**Authors:** Ming‑Yue Wang, Meng‑Fei An, Mao-Si Fan, Shao-Shi Zhang, Ze‑Rui Sun, Yun‑Li Zhao, Ze-Min Xiang, Jun Sheng

**Affiliations:** a Key Laboratory of Pu-erh Tea Science, Ministry of Education, Yunnan Agricultural University, Kunming, P. R. China; b College of Food Science and Technology, Yunnan Agricultural University, Kunming, P. R. China; c College of Science, Yunnan Agricultural University, Kunming, P. R. China; d Key Laboratory of Medicinal Chemistry for Natural Resource, Ministry of Education; Yunnan Provincial Center for Research & Development of Natural Products; School of Chemical Science and Technology, Yunnan University, Kunming, P. R. China; e State Key Laboratory for Conservation and Utilization of Bio-Resources in Yunnan, Kunming, P. R. China

**Keywords:** Ferulic acid ethyl ester, postmenopausal osteoporosis, osteoclast

## Abstract

**Context:**

Ferulic acid ethyl ester (FAEE) is abundant in *Ligusticum chuanxiong* Hort. (Apiaceae) and grains, and possesses diverse biological activities; but the effects of FAEE on osteoporosis has not been reported.

**Objective:**

This study investigated whether FAEE can attenuate osteoclastogenesis and relieve ovariectomy-induced osteoporosis via attenuating mitogen-activated protein kinase (MAPK).

**Materials and methods:**

We stimulated RAW 264.7 cells with receptor activator of NF-κB ligand (RANKL) followed by FAEE. The roles of FAEE in osteoclast production and osteogenic resorption of mature osteoclasts were evaluated by tartrate resistant acid phosphatase (TRAP) staining, expression of osteoclast-specific genes, proteins and MAPK. Ovariectomized (OVX) female Sprague-Dawley rats were administered FAEE (20 mg/kg/day) for 12 weeks to explore its potential *in vivo*, and then histology was undertaken in combination with cytokines analyses.

**Results:**

FAEE suppressed RANKL-induced osteoclast formation (96 ± 0.88 vs. 15 ± 1.68) by suppressing the expression of osteoclast-specific genes, proteins and MAPK signalling pathway related proteins (p-ERK/ERK, p-JNK/JNK and p-P38/P38) *in vitro*. In addition, OVX rats exposed to FAEE maintained their normal calcium (Ca) (2.72 ± 0.02 vs. 2.63 ± 0.03, *p* < 0.05) balance, increased oestradiol levels (498.3 ± 9.43 vs. 398.7 ± 22.06, *p* < 0.05), simultaneously reduced levels of bone mineral density (BMD) (0.159 ± 0.0016 vs. 0.153 ± 0.0025, *p* < 0.05) and bone mineral content (BMC) (0.8 ± 0.0158 vs. 0.68 ± 0.0291, *p* < 0.01).

**Discussion and conclusions:**

These findings suggested that FAEE could be used to ameliorate osteoporosis by the MAPK signalling pathway, suggesting that FAEE could be a potential therapeutic candidate for osteoporosis.

## Introduction

Bone is a dynamic organ (Casarrubios et al. 2016). Bone homeostasis is dependent upon the balance between bone resorption by osteoclasts and bone formation by osteoblasts (Tyagi et al. 2012). However, if more bone is resorbed than formed, then osteopenia, inferior bone architecture and ultimately, osteoporosis can occur (Zaidi 2007; Rachner et al. 2011).

Osteoporosis (OP) is a metabolic bone disease characterised by loss of bone mass and degeneration of bone microstructure, which leads to an increased risk of fracture (Chung et al. 2017; Kum et al. 2017). With ageing populations worldwide, osteoporosis is becoming a major public health problem worldwide estimated to affect 200 million people globally. Osteoporosis is found commonly in older people, especially postmenopausal women (Banu et al. 2012; Song et al. 2016). Oestrogen deficiency is a typical symptom after menopause in women (Wu et al. 2012). However, oestrogen can directly inhibit preosteoclasts differentiation and osteoclastogenesis directly (Janas and Folwarczna 2017), as well as promoting apoptosis of preosteoclasts and osteoclasts to decrease the number of osteoclasts (Lee et al. 2014). In addition, oestrogen stimulates osteoblasts to secrete osteoprotegerin (OPG), which can compete against receptor activator for NF-κB ligand (RANKL) and suppress osteoclastogenesis (Zhou et al. 2009; Tyagi et al. 2012).

Osteoclasts are multinucleated cells which differentiated from a monocyte/macrophage lineage haematopoietic precursor cells through a multi-stage process of proliferation, migration, fusion and activation (Boyle et al. 2003). The differentiation of osteoclasts requires two putative promoters of osteoclastogenesis RANKL and macrophage colony-stimulating factor (M-CSF) (Kong et al. 1999; Chen et al. 2013). Binding of RANKL to RANK recruits adaptor molecules called tumour necrosis factor receptor-associated factors (TRAFs), especially TRAF6, which leads to activation of mitogen-activated protein kinases (MAPKs), including extracellular signal-regulated kinase (ERK), c-Jun N-terminal kinase (JNK), and p38 (Darnay et al. 1998; Galibert et al. 1998; Boyle et al. 2003; Feng 2005). Moreover, changes in any one of these pathways can have profound effects on bone resorption and osteoclast differentiation (Teitelbaum and Ross 2003). Therefore, identification of agents that either inhibit osteoclast resorption or increase bone formation are vital for the development of novel drugs against osteoporosis.

Several regimens are available to treat osteoporosis, including vitamin D, hormone replacement therapy (HRT), selective oestrogen receptor modulators and bisphosphonates, but these treatments have side effects (Zhao et al. 2011; Li et al. 2016); For example, bisphosphonates can inhibit osteoclast resorption, and can damage the gastrointestinal tract (Pazianas and Abrahamsen 2011). Hormone replacement therapy (which is used commonly in postmenopausal women) can increase the risk of blood clots, cardiac arrest, breast cancer, and uterine cancer, and its long-term use is prohibited (Qiu et al. 2007). Hence, developing efficacious and safe strategies for the prevention and treatment of osteoporosis is needed.

**Figure 1.**
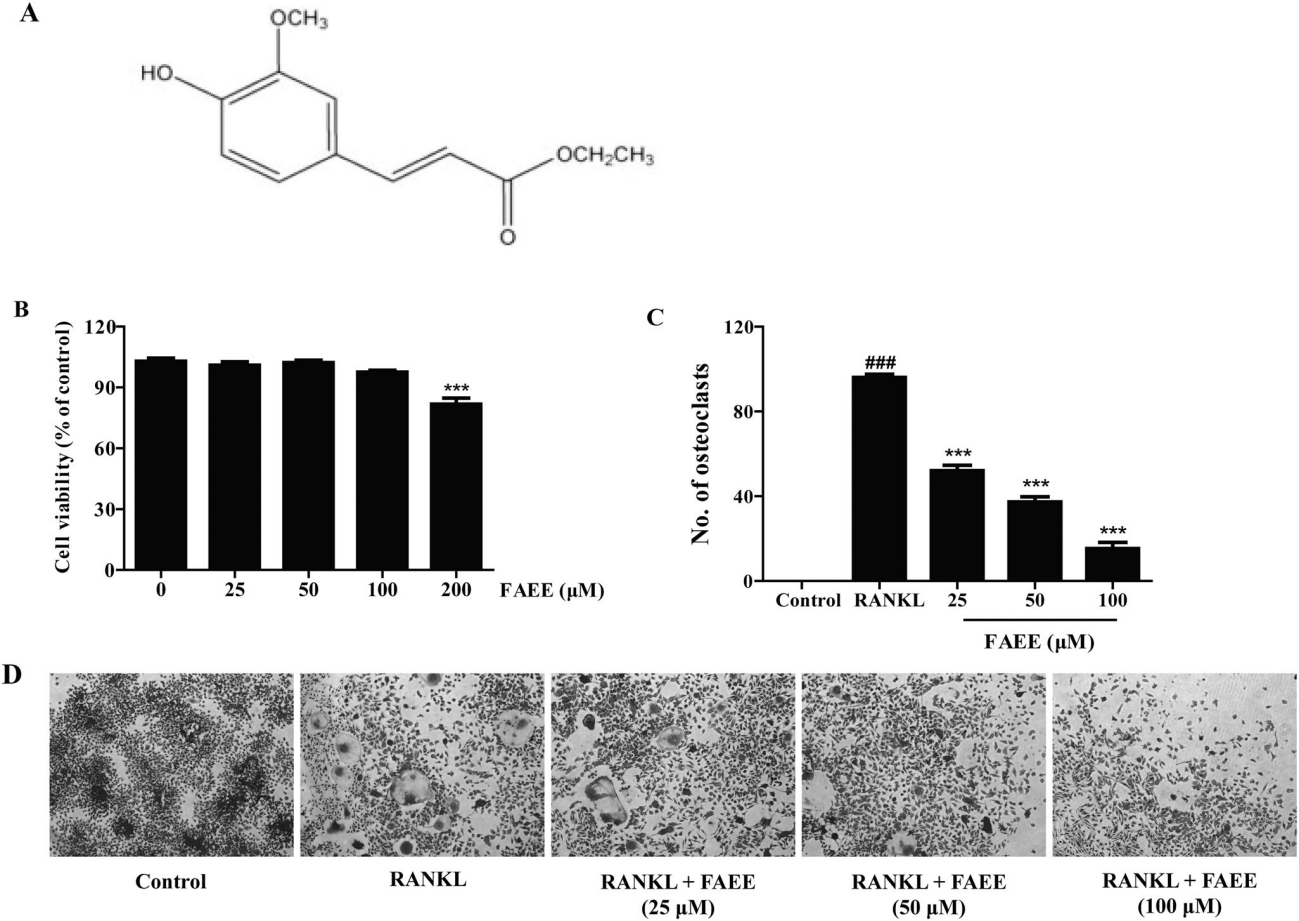
Effect of FAEE on cell viability and osteoclast formation. The chemical structure of ferulic acid ethyl esther (ethyl 4-hydroxy-3-methoxycinnamate) (A). RAW 264.7 cells were exposed to various concentrations of FAEE (25, 50, 100, or 200 μM) for 48 h and the (4,5-dimethylthiazol-2-yl)-2,5-diphenyltetrazolium bromide (MTT) assay was employed to evaluate cell viability(B). RAW 264.7 cells were incubated with FAEE (25, 50, or 100 μM) in the presence of receptor activator of NF-κB ligand (RANKL) (50 ng/mL) for 5 days. Tartrate resistant acid phosphatase (TRAP)-positive cells that had ≥3 nuclei were identified as osteoclasts and quantified using light microscopy (C). TRAP staining with representative images of RAW 264.7 cells (D). Data are the mean ± SEM of triplicate experiments. ### *p* < 0.001 versus RAW 264.7 cells without RANKL, *** *p* < 0.001 versus RAW 264.7 cells with RANKL alone.

Ferulic acid ethyl ester (ethyl 4-hydroxy-3-methoxycinnamate (FAEE)) (Figure 1(A)) is a natural ester philic derivative of ferulic acid (FA), which is a monomeric component purified from traditional Chinese medicinal herb *Ferula* (Wang et al. 2021) and widely present in natural plants such as *Ligusticum chuanxiong* Hort. (Apiaceae), *Ferula* species, and in grains (e. g., maize and rice) (Zhang et al. 2010; Sultana 2012). Moreover, FAEE has more lipophilic than FA and retains the characteristic low toxicity of FA (Wang et al. 2008; 2021). FAEE has a wide range of pharmacological properties including anti-inflammatory (Bolling et al. 2011; Cunha et al. 2016), anti-atherosclerosis (Tsai et al. 2015), antithrombosis (Wang 2002), antitumor (Li et al. 2012) effects, and can protect the liver (Wang et al. 2004b), and vascular endothelial cells (Wang et al. 2004a). As its beneficial health properties, FAEE has been widely studied.

Scholars have demonstrated that inflammation is a feature of osteoporosis (Chen et al. 2017). Proinflammatory cytokines increase bone resorption by inducing osteoclast differentiation, inhibiting osteoblast maturation and, therefore, breaking the dynamic balance of bone homeostasis and accelerating bone loss (Maruotti et al. 2011; Adamopoulos and Mellins 2015). In addition, FAEE has been demonstrated to exert an inhibitory effect on the MAPK signalling pathway *in vivo* and *in vitro* (Phuong et al. 2014). Thus, we hypothesised that FAEE could possess anti-osteoporotic activity and be a promising candidate for development of anti-osteoporosis drugs. We explored the effect of FAEE on RANKL-induced osteoclast differentiation in RAW 264.7 cells and on an ovariectomy-induced osteoporosis model in rats, and investigated the potential mechanism.

## Materials and methods

### Reagents and antibodies

FAEE was purchased from (Kramar) Shanghai Pichen Biotechnology Co., Ltd. (Shanghai, China). The murine macrophage cells line (RAW 264.7) was obtained from American Type Culture Collection (Manassas, VA, USA). Dulbecco’s modified Eagle’s medium (DMEM) and foetal bovine serum (FBS) were purchased from Thermo Fisher Biochemical Products (Beijing) Co., Ltd. (Beijing, China) and Biological Industries (Kibbutz Beit Haemek, Israel), respectively. Staining kits for the 3-(4,5-dimethylthiazol-2-yl)-2,5-diphenyltetrazolium bromide (MTT) assay and TRAP were obtained from Sigma-Aldrich (Saint Louis, MO, USA). *Escherichia coli*-derived recombinant mouse RANKL was purchased from R&D Systems (Minneapolis, MN, USA). Polymerase chain reaction (PCR) primer pairs were obtained from Generay Biotechnology (Shanghai, China). The mixed penicillin and streptomycin solution (P/S) and Rhodamine-conjugated phalloidin were obtained from Solarbio (Beijing, China). Antibodies against nuclear factor of activated T cells c1 (NFATc1), c-Fos and c-Src were obtained from Santa Cruz Biotechnology (Santa Cruz, CA, USA). Anti-β-tubulin was bought from Proteintech (Rosemont, IL, USA). Horseradish peroxidase (HRP)-conjugated goat anti-mouse and anti-rabbit immunoglobulin (Ig)G were acquired from R&D Systems (Minneapolis, MN, USA). Antibodies against phosphorylated (p)-ERK1/2 (Thr202/Thy204), ERK, p-JNK (Thr183/Thy185), JNK, p-P38 (Thr180/Thy182), and P38 were supplied by Cell Signalling Technology (Danvers, MA, USA). Alkaline phosphatase (ALP) and Ca assay kits were bought from Ningbo Ruiyuan Biotechnology (Ningbo, China). Enzyme linked immunosorbent assay (ELISA) kits for C-terminal telopeptide of type I collagen (CTX-I), oestrogen, and Interleukin-1β (IL-1β) were obtained from Wuhan Huamei Biotechnology (Wuhan, China).

### Cell viability assay

RAW 264.7 cells were incubated in DMEM supplemented with 10% FBS and 1% P/S at 37 °C in an atmosphere of 5% CO_2_. We measured the cell viability of RAW 264.7 cells after FAEE treatment using the MTT assay. Briefly, cells were seeded on 96-well plates at a density of 2 × 10^4^/well and incubated overnight. Cells were exposed to FAEE (25, 50, 100, or 200 μM) for an additional 48 h. After incubation for a specified time, 20 μL of MTT was added into per well and incubation allowed for an additional 4 h. Then, cells supernatants were removed followed by addition 200 μL of dimethyl sulfoxide to dissolve formazan crystals. Absorbance was measured at 492 nm with a FlexStation^TM^ 3 Multi-Mode Microplate Reader (Molecular Devices, Silicon Valley, CA, USA). Results are expressed as a percentage of the control.

### Osteoclastogenesis assay in vitro

96-well plates were used to culture RAW 264.7 cells at 2 × 10^3^ cells/well overnight. The following day, FAEE (25, 50, or 100 μM) was used to pretreat cells for 30 min, and then cells were stimulated with of RANKL (50 ng/mL) for another 5 days. Then, plates were fixed with fixative solution by combining Citrate Solution, acetone and 37% formaldehyde and staining with TRAP according to manufacturer instructions. TRAP-positive cells that contained >3 nuclei were regarded as mature osteoclasts and counted using a light microscope.

### Formation of F-actin rings and bone pit-formation assay

RAW 264.7 cells were seeded on 12-well plates (5 × 10^3^ cells/well). Cells were cultured for 24 h and pre-treated with FAEE (25, 50, or 100 μM) in combination with RANKL (50 ng/mL). 5 days after treatment, cells were fixed with 4% paraformaldehyde for 10 min, washed thrice with phosphate buffered saline (PBS), and then permeabilized with 0.5% Triton-X-100 for 5 min. Next, cells were stained with rhodamine phalloidin (100 nM) for 30 min at room temperature (4-37 °C) and in dark. After washing thrice in PBS, plates were treated with mounting medium containing 4,6-diamidino-2-phenylindole (DAPI) and a cover glass placed over them. The immunofluorescent staining sheet was observed and images captured using a confocal laser microscope (DM 2000; Leica, Wetzlar, Germany).

RAW 264.7 cells were cultured in Osteo Assay Surface multi-well plates (Corning, Corning, NY, USA) at 2.5 × 10^3^ cells/well and incubated for 24 h. Cells were pre-treated with FAEE (25, 50, or 100 μM) for 30 min followed by RANKL (50 ng/mL) for 5 days. Samples were washed with PBS and dried for 3-5 h in air. Photomicrographs of the resorbed areas were captured using a light microscope.

### RNA isolation and gene expression analysis

Total cellular RNA was isolated from cultured RAW 264.7 cells by TransZol Up (Trans Gen Biotech, Beijing, China). Complementary DNA (cDNA) was reverse-transcribed from total RNA (1 μg) with the PrimeScript RT Reagent Kit with a genomic DNA Eraser (Takara Biotechnology, Shiga, Japan). Real-time quantitative polymerase chain reaction (qRT-PCR) was undertaken with an ABI PRISM® 7900 HT Fast Real-Time PCR system (Applied Biosystems, Foster City, CA, USA) using SYBR™ Premix ExTaq^TM^ II (Takara Biotechnology). The relative standard curve method (2^-ΔΔCt^) was applied to determine relative gene expression, and the result was normalised based on expression of the endogenous gene GAPDH. The PCR primers used in this experiment are listed below: mouse *GAPDH*, 5′-AACTTTGGCATTGTGGAAGG-3′ (forward) and 5′-ACACATTGGGGGTAGGAACA-3′ (reverse); *TRAP,* 5′-GCTGGAAACCATGATCACCT-3′ (forward) and 5′- GAGTTGCCACACAGCATCAC-3′ (reverse); *c-Src*, 5′-CCAGGCTGAGGAGTGGTACT-3′ (forward) and 5′-CAGCTTGCGGATCTTGTAGT-3′ (reverse); *Cathepsin K*, 5′-CTTCCAATACGTGCAGCAGA-3′ (forward) and 5′-TCTTCAGGGCTTTCTCGTTC-3′ (reverse); *NFATc1*, 5′-TGGAGAAGCAGAGCACAGAC-3′ (forward) and 5′-GCGGAAAGGTGGTATCTCAA-3′ (reverse); *MMP-9*, 5′-CGTCGTGATCCCCACTTACT-3′ (forward) and 5′-AACACACAGGGTTTGCCTTC-3′ (reverse); *OSCAR*, 5′-GGGGTAACGGATCAGCTCCCCAGA-3′ (forward) and 5′-CCAAGGAGCCAGAACGTCGAAACT-3′ (reverse); *Acp-5*, 5′-CTGGAGTGCACGATGCCAGCGACA-3′ (forward) and 5′-TCCGTGCTCGGCGATGGACCAGA-3′ (reverse); *Atp6v0d2*, 5′-GAAGCTGTCAACATTGCAGA-3′ (forward) and 5′-TCACCGTGATCCTTGCAGAA-3′ (reverse).

### Western blotting

RAW 264.7 cells were seeded on 60 mm plates (5.5 × 10^5^ cells/plate) and incubated overnight. Cells were pre-incubated with FAEE (25, 50, or 100 μM) for 30 min and then stimulated with RANKL (50 ng/mL) for 48 h. Extracted protein was used to analyse the effect of FAEE on expression of the osteoclast-related markers c-Fos, c-Src and NFATc1 by western blotting. Also, RAW 264.7 cells were seeded into 60 mm plates at 2.2 × 10^6^ cells/plate overnight for 4 h to explore the effects of FAEE (100 μM), and then RANKL (50 ng/mL) was added at the indicated time (0, 15, 30 or 60 min). Thereafter, cells were washed with cold PBS and lysed by a radioimmunoprecipitation (RIPA) lysis solution (Solarbio) on ice. Extracted proteins were quantified by a bicinchoninic acid (BCA) assay. Equal amounts of protein (40 μg) were separated by sodium dodecyl sulfate-polyacrylamide gel electrophoresis using 10% gels. The proteins on gels were transferred onto polyvinylidene fluoride (PVDF) membranes; (Millipore, Waltham, MA, USA). Immunoblots were blocked with 5% defatted milk in TBST (Tris-buffered saline (TBS) containing 0.1% Tween 20) buffer at room temperature for 1 h to reduce non-specific binding. PVDF membranes were cultured overnight at 4 °C with appropriate primary antibodies (1:1000 dilution) specific to c-Fos, c-Src, NFATc1, p-ERK1/2, ERK, p-JNK, JNK, p-P38 and P38, washed thrice with TBST and incubated with horseradish peroxidase-conjugated secondary goat anti-mouse or anti-rabbit antibodies for 1 h. Images of the blots were obtained with a FluorChem E System (ProteinSimple, Santa Clara, CA, USA).

### Animal experiment

Specific pathogen-free female Sprague-Dawley rats (220-250 g) were bought from Kunming Medical University (license number: SCXK K2015-0002). Rats were housed in ventilated filter-top cages and exposed to a 12 h light/dark cycle at 24 ± 1 °C and 50-60% humidity. Rats were allowed to acclimatise to their environment for 7 days and fed standard rat chow and water *ad libitum* before experimentation. All experimental procedures were carried out according to the guidelines of the Institutional Animal Care and Use Committee of the Yunnan Agricultural University (approval number: YNAU2019LLWYH003-1). All rats were anaesthetised with isoflurane by inhalation. An OVX rat model was produced by removing bilateral ovaries from rats. Sham-operated rats were created by removing the same amount of adipose tissues, as described previously (Wang et al. 2018).

Seven days after ovariectomy, rats were divided randomly into the three groups of eight according to their weight: model (OVX rats treated with distilled water); positive control [OVX rats treated with oestradiol-valerate tablets (E_2_) (1 mg/kg)]; OVX + FAEE [OVX rats treated with FAEE (20 mg/kg)]. Treatment group rats were administered FAEE or an equal volume of distilled water once a day by gavage. Then, 12 weeks after treatment, all rats were fasted (except water consumption) overnight. Blood was collected from the femoral artery of rats in sham, model, OVX + E_2_, and OVX + FAEE groups and centrifuged for 10 min at 4000 rpm at 4 °C to extract serum. The left femur, right femur and uterus of all rats were excised and weighed immediately, and preserved in 10% neutral formaldehyde for subsequent histopathology.

### Bone mineral density (BMD) and bone mineral content (BMC)

Before the first two weeks of anatomisation, all rats were narcotised with isoflurane and measured BMD and BMC of left femur with dual energy X-ray bone densitometry (Lunar Prodigy Advance DEXA, GE Healthcare, Madison, WI, USA) by scanning mode of small laboratory animals (Pastoureau et al. 1995). Measurements for the whole body and left femur of rats were calculated automatically with Encore 2006 (GE Healthcare, Madison, WI, USA).

### Serum levels of biochemical indicators and the organ coefficient

Serum levels of Ca^+2^ and ALP were measured using an automatic analyser (AU 680; Beckman Coulter, Fullerton, CA, USA). Oestrogen levels and levels of the proinflammatory cytokines IL-1β were measured using ELISAs in accordance with manufacturer instructions.

After euthanasia, the uterus and femurs of rats were collected after removing the surrounding adhesions and soft tissues. The weights of the uterus and femurs was measured in milligrams and grams to determine the wet weights. The organ coefficient was calculated using the following formula: organ coefficients = wet weight (mg or g)/bodyweight (g) × 100.

### Bone histopathology

Sections of the right femur of rats underwent histopathology. After tissues had been fixed with 10% neutral formaldehyde for 72 h, they were flushed with running water for 4 h, and dehydrated using a graded series of ethanol solutions. These actions were followed by dewaxing with xylene, embedding with paraffin, and cutting into sections of thickness 5 μm. These sections were processed for haematoxylin and eosin (H&E) staining.

### Statistical analysis

Data are the mean ± standard error of the mean (SEM). Each experiment was repeated three times. Data were assessed with SPSS 17.0 (IBM, Armonk, NY, USA) and Prism 5 (Graph Pad, San Diego, CA, USA) by one-way ANOVA followed by a two-tailed Student’s *t*-test. Significance was set at *p* < 0.05.

## Results

### Effect of FAEE on cell viability and osteoclastogenesis

To investigate the effects of FAEE on cell viability *in vitro*, we first exposed RAW 264.7 cells to FAEE (25, 50, 100, or 200 μM) for 48 h. The viability of RAW 264.7 cells decreased upon treatment with FAEE at 200 μM (*p* < 0.001) (Figure 1(B)). Thus, FAEE at 25, 50, and 100 μM was chosen for further testing.

To investigate the effect of FAEE on osteoclastogenesis, we established a standard model of osteoclast differentiation *in vitro*: the osteoclast precursor cell line, RAW 264.7, was induced by RANKL (50 ng/mL) treatment. When the osteoclast precursor cells differentiated on day-5, many osteoclasts were formed and evaluated with the TRAP staining kit. Addition of FAEE (25, 50, or 100 μM) suppressed TRAP-positive multinucleated osteoclasts in a dose-dependent manner in our osteoclast-differentiation model (*p* < 0.001) (Figure 1(C,D)).

### FAEE inhibits RANKL-induced formation of F-actin rings and bone resorption

**Figure 2.**
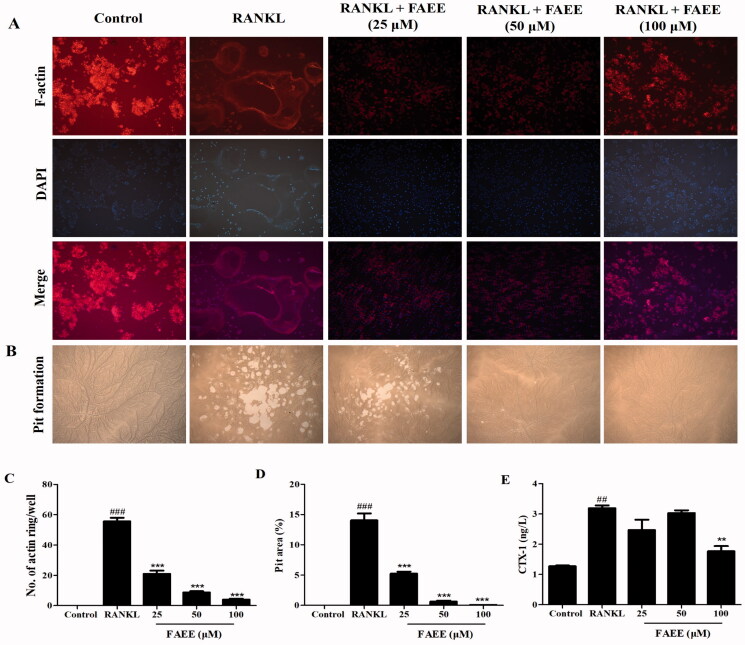
Effect of FAEE on RANKL-stimulated osteoclast formation of F-actin rings in osteoclasts and bone resorption. In the assay to measure formation of F-actin rings, RAW 264.7 cells were plated into 12-well plates and incubated with FAEE (25, 50, or 100 μM) in the presence of RANKL for 5 days. Cells were fixed and stained with fluorescein rhodamine phalloidin to observe F-actin rings (magnification: 200×) and were merged by Image J software (A), the number of F-actin rings were calculated under a fluorescence microscope (C). In the pit-formation assay, RAW 264.7 cells were seeded on an Osteo Assay Surface multi well plates. Surfaces were treated similarly and cultured for 5 days. Resorption area was captured using an inverted microscope (magnification: 100×) (B). Pit areas were quantified by Image J software (D). C-terminal telopeptide of type I collagen (CTX-I) concentration in cell supernatants using Enzyme-linked immune-sorbent assay (ELISA) (E). Data are the mean ± SEM of triplicate experiments. ## *p* < 0.01 and ### *p* < 0.001 versus RAW 264.7 cells without RANKL, ** *p* < 0.01 and *** *p* < 0.001 versus RAW 264.7 cells with RANKL alone.

We explored how FAEE affected RANKL-stimulated formation of F-actin rings in osteoclasts. This phenomenon is a prerequisite for osteoclast bone resorption and the most arresting feature of mature osteoclasts during osteoclast formation. RAW 264.7 cells differentiated into mature osteoclasts and formed obvious F-actin rings in the presence of RANKL (Figure 2(A)). Nevertheless, the number and size of F-actin rings was reduced markedly if cells were incubated with FAEE (25, 50, or 100 μM) (*p* < 0.001) (Figure 2(C)). These results indicated that FAEE suppressed osteoclastogenesis through inhibition of the formation of F-actin rings.

To further assess if FAEE inhibited osteoclast bone resorption by osteoclasts, we examined the effects of FAEE on the bone resorbing activity on a bone biomimetic synthetic surface. Many resorption pits were formed on the plates in the RANKL group compared with that for cells cultured without RANKL. However, osteoclast activity was impaired obviously by FAEE treatment (25, 50, or 100 μM), as illustrated by fewer resorption pits (Figure 2(B)). The statistical analysis explained that FAEE inhibited the bone pit area of osteoclast in a dose-proportional manner compared to that in the RANKL group (*p* < 0.001) (Figure 2(D)).

CTX-I is a marker of bone resorption. The CTX-I level in cell supernatants after treatment with RANKL alone was increased significantly compared with that in the control group (*p* < 0.01) (Figure 2(E)). Nevertheless, treatment with FAEE (100 μM) decreased the CTX-I level significantly compared with that in the RANKL group (*p* < 0.01).

### FAEE suppressed expression of RANKL-induced osteoclast-specific genes and proteins

**Figure 3.**
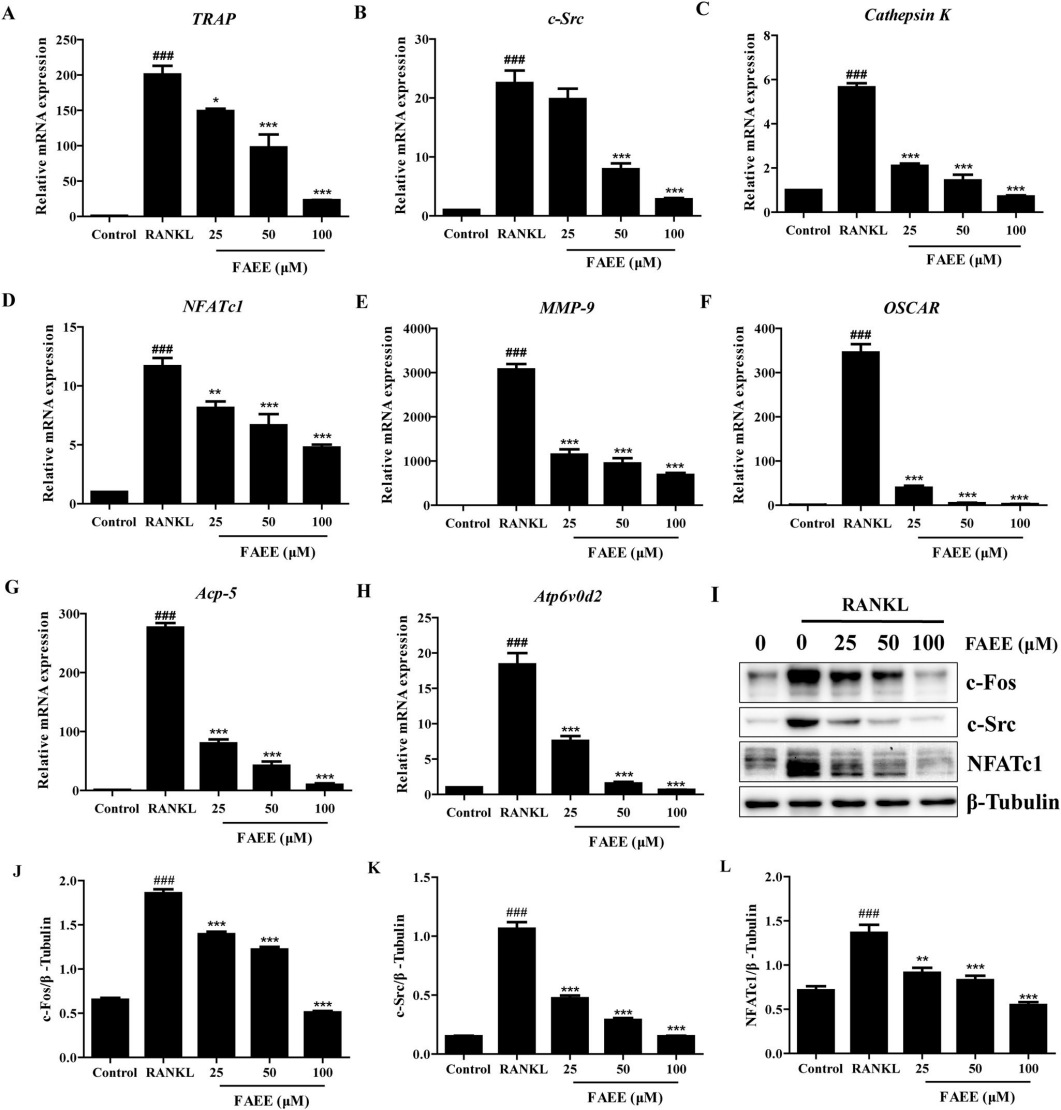
Effect of FAEE on the expression of osteoclastogenesis-associated genes and proteins (A-I). FAEE inhibited RANKL-induced mRNA expression of TRAP (A), c-Src (B), Cathepsin K (C), Nuclear factor of activated T cells c1 (NFATc1) (D), Matrix metalloproteinase 9 (MMP-9) (E), The osteoclast-associated receptor (OSCAR) (F), Acp-5 (G), and Atp6v0d2 (H) in RAW 264.7 cells as determined by qRT-PCR. FAEE inhibited RANKL-induced protein expression of c-Fos, c-Src and NFATc1 (I) as determined by western blotting. Quantitative ratio of c-Fos (J), c-Src (K) and NFATc1 (L) to the band strength of β-Tubulin. Data are the mean ± SEM of triplicate experiments. ### *p* < 0.001 versus RAW 264.7 cells without RANKL, ** *p* < 0.01 and *** *p* < 0.001 versus RAW 264.7 cells with RANKL alone.

Osteoclast differentiation and bone resorption result in up-regulation of expression of osteoclast specific genes and proteins expression which, in turn, are stimulated by RANKL. We explored if FAEE inhibited expression of osteoclast-specific genes and proteins. FAEE treatment resulted in the suppression of the RANKL-induced mRNA expression of *TRAP*, *c-Src*, *cathepsin K*, *NFATc1*, *matrix metalloproteinase* (*MMP)-9*, the *osteoclast-associated receptor (OSCAR)*, *Acp-5* and *Atp6v0d2* (*p* < 0.01, *p* < 0.05 and *p* < 0.001, respectively) (Figure 3(A–H)), and protein expression of c-Fos, c-Src and NFATc1, in a dose-dependent manner (*p* < 0.001) (Figure 3(I–L)). These findings suggested an inhibitory effect of FAEE on osteoclastogenesis and bone resorption.

### FAEE suppressed RANKL-stimulated activation of the MAPK pathway

**Figure 4.**
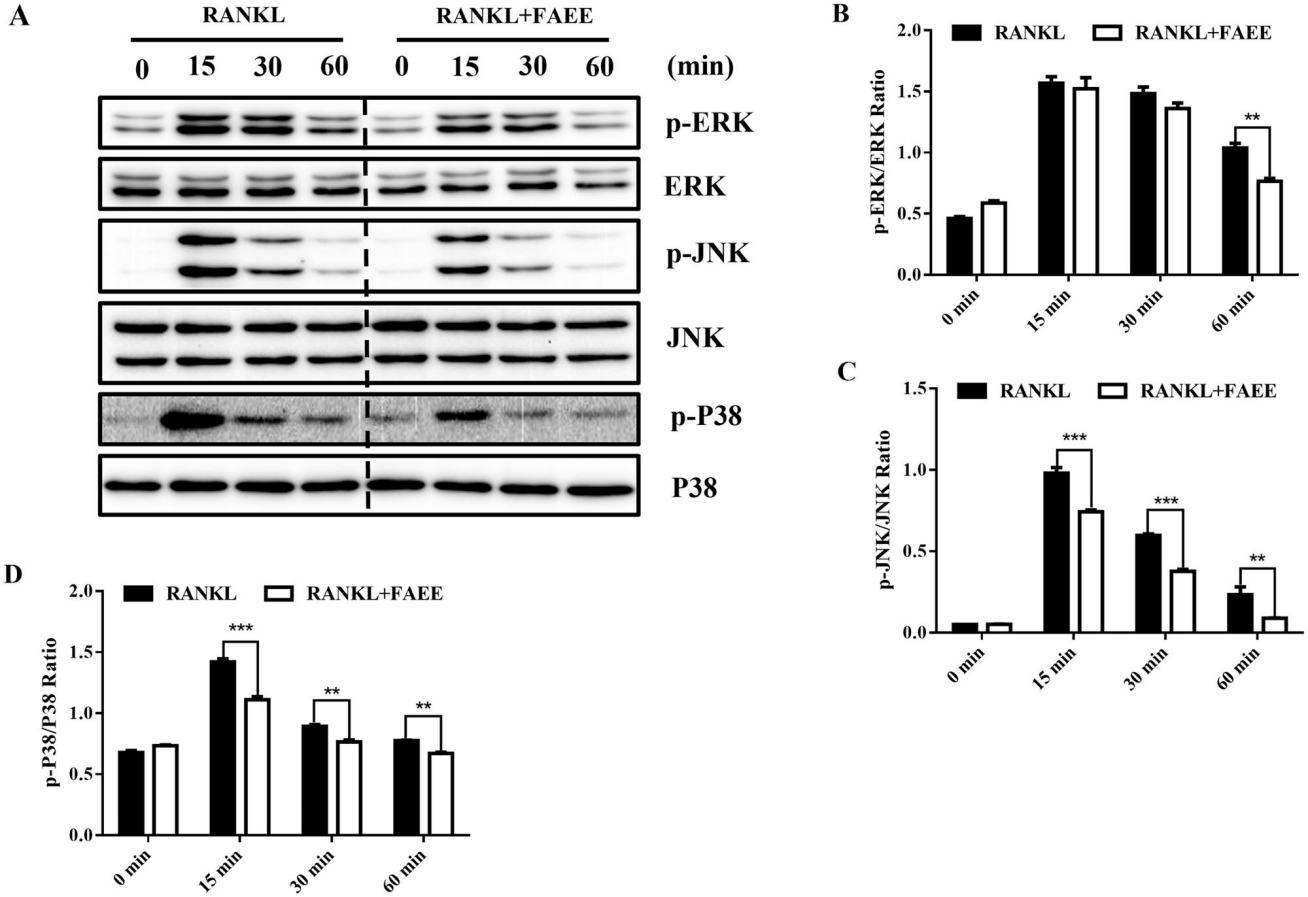
Effect of FAEE on the RANKL-induced mitogen-activated protein kinase (MAPK) signalling pathway during osteoclastogenesis. RAW 264.7 cells were incubated with or without FAEE (100 μM) for 4 h, and then stimulated with RANKL (50 ng/mL) for the indicated times. Then, cell lysates were extracted and underwent western blotting with antibodies as indicated. FAEE blocked the phosphorylation of the extracellular signal-regulated kinase (ERK), the Jun N-terminal kinase (JNK) and p38 induced by RANKL. Representative Western Blot images of the effect of FAEE on the RANKL-induced MAPK signalling pathway (A). The ratios of phosphorylated ERK, JNK, and P38 relative to total ERK (B), total JNK (C), and total P38 (D) were quantified by Alphaview Software (Cell Biosciences, Santa Clara, CA, USA). Data are the mean ± SEM of triplicate experiments. ### *p* < 0.001 versus RAW 264.7 cells without RANKL, ** *p* < 0.01 and *** *p* < 0.001 versus RAW 264.7 cells with RANKL alone.

Activation of the MAPK pathway plays an important part in osteoclastogenesis. We found that FAEE inhibited RANKL-induced osteoclast formation. We evaluated the effects of FAEE on the MAPK pathway after incubation with RAW 264.7 cells in the presence of RANKL. We investigated the phosphorylation of three major subfamilies of MAPKs (ERK, cJNK and p38) by western blotting. Phosphorylation of these three major subfamilies reached its peak within 60 min of RANKL stimulation (Figure 4(A)). FAEE treatment inhibited the phosphorylation of ERK (*p* < 0.01) (Figure 4(B)), JNK (*p* < 0.01 and *p* < 0.001, respectively) (Figure 4(C)) and p38 (*p* < 0.01 and *p* < 0.001, respectively) (Figure 4(D)) significantly compared with that in the RANKL-evoked control. These results suggested that FAEE could inhibit RANKL-stimulated activation of the MAPK pathway in osteoclasts.

### Effect of FAEE on BMC and BMD in OVX rats

**Figure 5.**
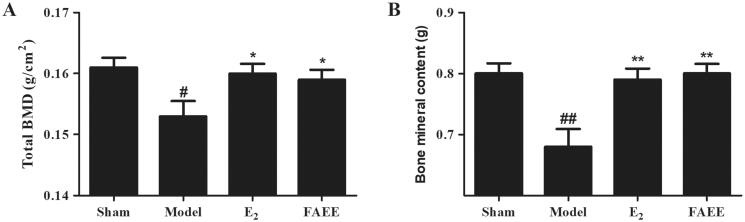
Effect of FAEE treatment on bone mineral density (BMD) and bone mineral content (BMC) in OVX rats. BMD (A) and BMC of the left femur (B). Data are the mean ± SEM of triplicate experiments. # *p* < 0.05, ## *p* < 0.01 versus sham-operated rats, * *p* < 0.05, ** *p* < 0.01 versus OVX rats.

To elucidate the effect of the FAEE treatment on OVX-evoked osteoporotic rats, we evaluated total-body BMD and left femur BMC with dual-energy X-ray absorptiometry (DEXA). A marked decline in BMD and BMC was noted when OVX rats were compared with sham-operated rats (*p* < 0.05 and *p* < 0.01, respectively) (Figure 5(A,B)). Treatment of OVX rats with E_2_ or FAEE significantly prevented the OVX-induced the decrease in bone parameters (*p* < 0.01 and *p* < 0.05, respectively).

### FAEE affects the serologic markers of osteoclasts and the organ coefficients in OVX rats

**Figure 6.**
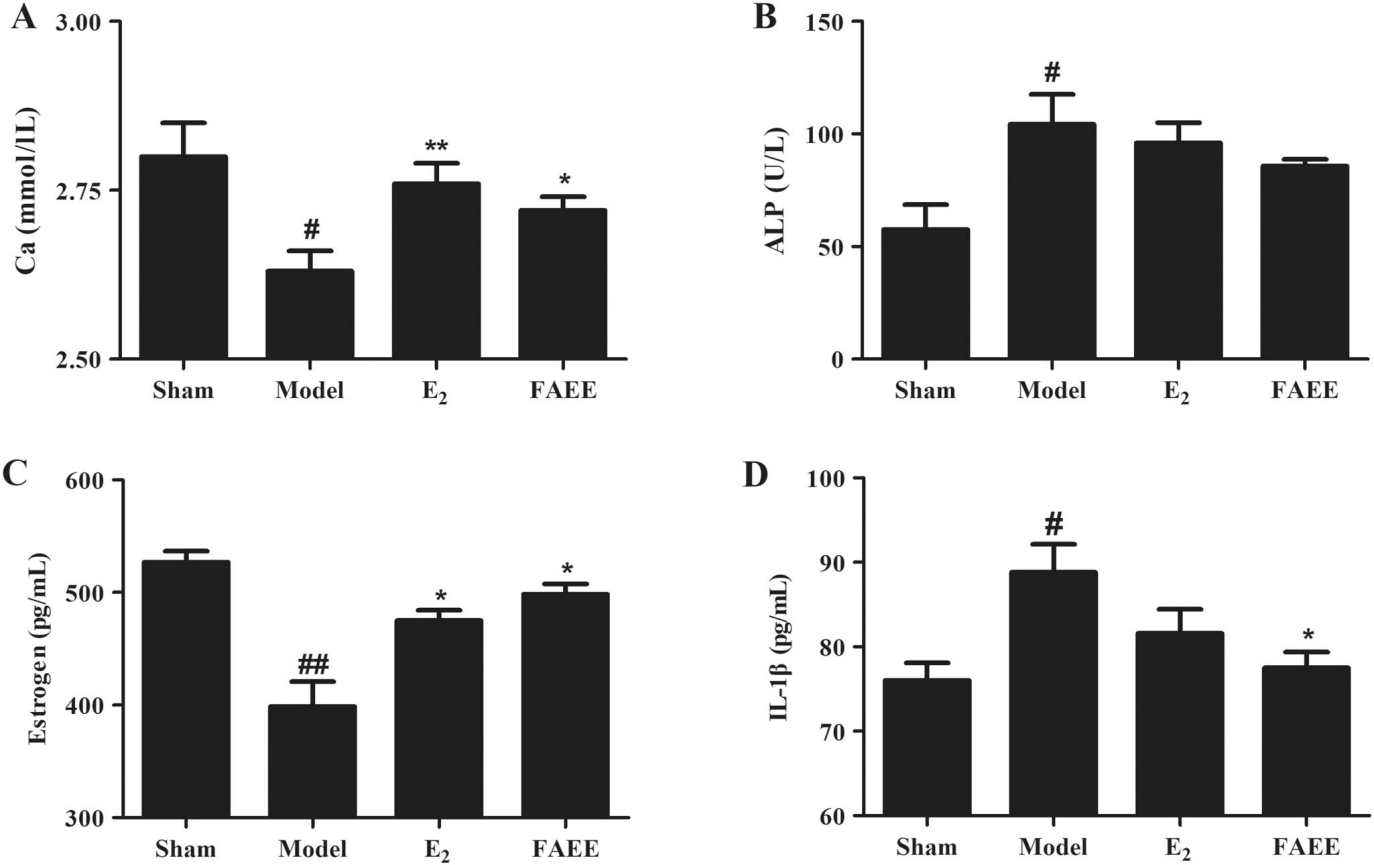
Effect of FAEE treatment on serum biochemical indicators in OVX rats. Calcium (Ca) (A), alkaline phosphatase (ALP) (B), oestrogen (C), and interleukin-1β (IL-1β) (D). Data are the mean ± SEM of triplicate experiments. # *p* < 0.05, ## *p* < 0.01 versus sham-operated rats, * *p* < 0.05, ** *p* < 0.01 versus OVX rats.

We investigated the effects of FAEE on serologic indicators and the organ coefficients of the uterus and femur using an OVX model in rats. The serum level of Ca was lower in OVX rats than that in sham rats (*p* < 0.05) (Figure 6(A)). However, the serum Ca^+2^ level in the OVX + E_2_ (positive control) group and FAEE group was recovered significantly compared with that in the OVX group (*p* < 0.01 and *p* < 0.05, respectively). The ALP level in the model group was higher than that in the sham group (*p* < 0.05), whereas there was a significant downward trend in the OVX + E_2_ and FAEE group (*p* > 0.05) (Figure 6(B)).

OVX rats had reduced oestrogen levels, so we investigated if FAEE could improve oestrogen levels in OVX rats. The serum oestrogen level was reduced in OVX rats compared with that in sham operated rats (*p* < 0.01). As expected, the OVX + E_2_ group and FAEE group had increased serum oestrogen levels compared with those in the model group (*p* < 0.05) (Figure 6(C)).

Proinflammatory cytokines in serum affect the function and formation of osteoclasts through different mechanisms. We measured IL-1β secretion in rat serum. The serum IL-1β level in OVX rats was significantly higher than that in sham-operated rats (*p* < 0.05); The OVX + E_2_ group showed a downward trend (*p* > 0.05) and FAEE treatment decreased the serum IL-1β levels compared with that in the model group (*p* < 0.05) (Figure 6(D)). These findings indicated that FAEE treatment could ameliorate bone homeostasis in OVX rats.

**Figure 7.**
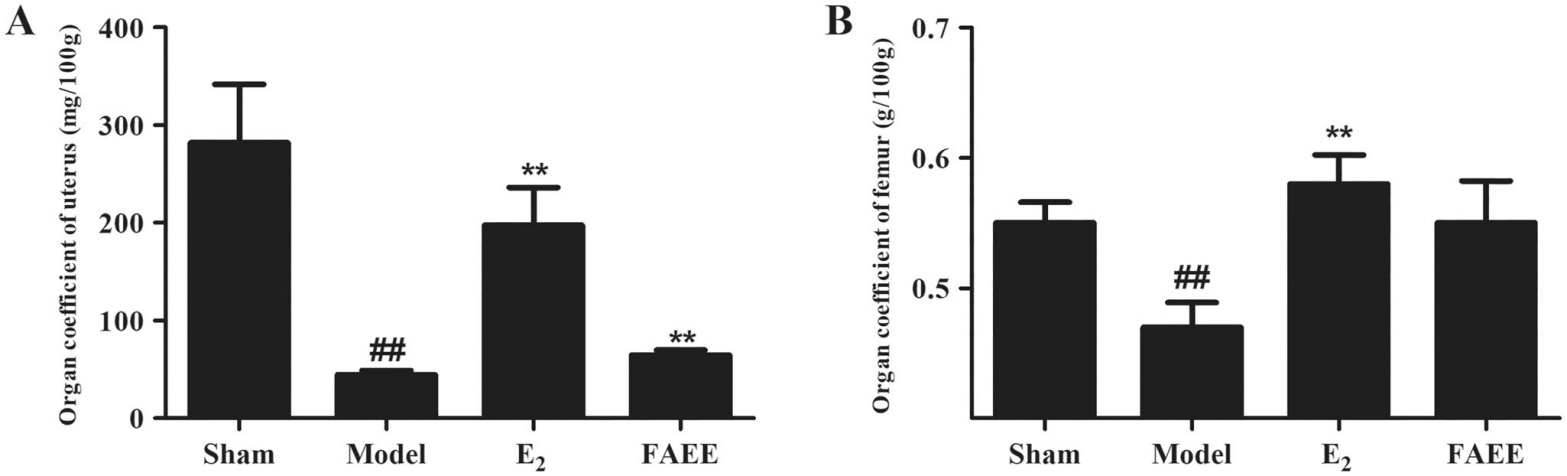
Effect of FAEE treatment on the organ coefficient in OVX rats. Uterus (A) and femur (B). Data are the mean ± SEM of triplicate experiments. ## *p* < 0.01 versus sham-operated rats, ** *p* < 0.01 versus OVX rats.

The organ coefficients for the uterus and femur were reduced markedly in OVX rats (*p* < 0.01) (Figure 7(A,B)), and treatment with E_2_ increased it markedly, compared with that in the model group (*p* < 0.01). However, FAEE treatment increased in the organ coefficients for the uterus compared with that in the model group (*p* < 0.01). FAEE treatment did not elicit a significant difference in the organ coefficients for the femur, but an improving trend was noted compared with that in OVX rats (*p* > 0.05).

### Effect of bone disease in OVX rats

**Figure 8.**
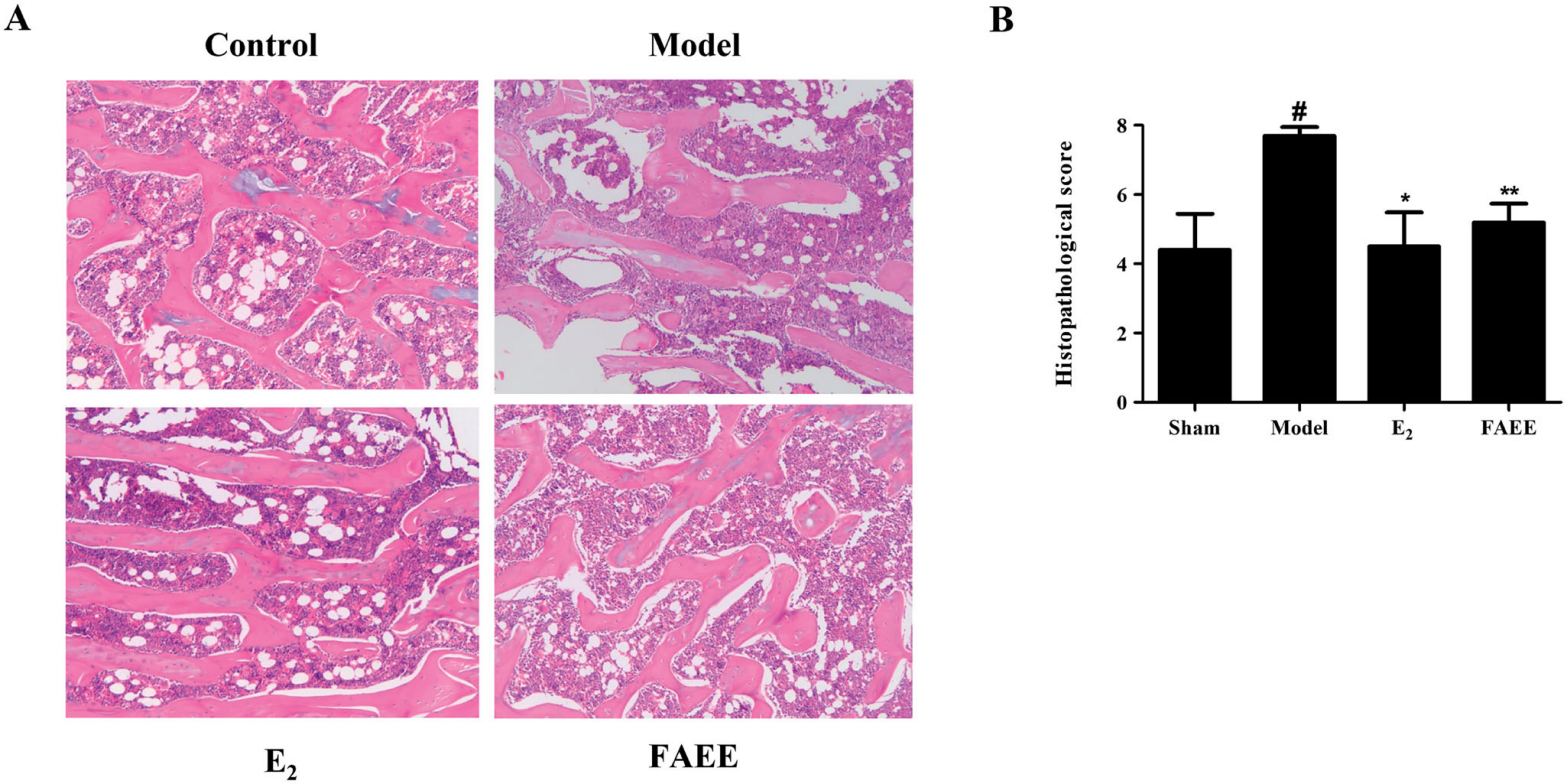
Effect of FAEE treatment on bone histopathology in OVX rats. Haematoxylin and eosin (H&E) staining showing representative images of the right femur (A) and histology scores for the left femur (B) in OVX rats. Data are the mean ± SEM of triplicate experiments. # *p* < 0.05, versus sham-operated rats, * *p* < 0.05, ** *p* < 0.01 versus OVX rats.

We undertook histology of the cortical bone and trabecular bone of the right femur in OVX rats. H&E staining revealed that the cortical bone thickness and trabecular area of rats in the sham-operated group remained normal, but OVX rats had significantly lower cortical bone thickness and trabecular area than those in the model group (Figure 8(A)). Moreover, the Histological Score for the femur was increased markedly in the model group compared with that in the sham-operated group (*p* < 0.05); whereas treatment with E_2_ or FAEE reduced significantly compared with that in the model group (*p* < 0.01 and *p* < 0.05) (Figure 8(B)). These results demonstrated that FAEE could improve bone loss during osteoporosis.

## Discussion

The present study suggested that FAEE has anti-osteoporotic effects in the RANKL-induced differentiation of RAW 264.7 cells and an ovariectomy-induced osteoporosis model in rats. This is the first study to demonstrate that the MAPK signalling pathway is involved in the bone-protective effects of FAEE against osteoporosis.

RANKL is a factor for osteoclast differentiation and belongs to a member of the tumour necrosis family, which promotes osteoclast differentiation (Kwan Tat et al. 2004; Pengjam et al. 2016). RAW 264.7 cells are a commonly used osteoclast precursor lines that can differentiate into osteoclasts induced by RANKL (Collin-Osdoby and Osdoby 2012). TRAP is regarded as a mark of the osteoclast phenotype, which is also used commonly to identify the differentiation of osteoclast precursors into osteoclasts (Kim et al. 2019). Thus, TRAP staining is a standard method for detecting osteoclast differentiation (Hayman 2008). In addition, mature osteoclasts express the TRAP, and it forms F-actin rings and causes bone resorption. Formation of F-actin rings and osteoclastic bone resorption are the most evident features of mature osteoclasts (Marchisio et al. 1984; Kim et al. 2020). In the present study, RANKL not only differentiated pre-osteoclastic RAW 264.7 cells into osteoclasts, it also increased the number of F-actin rings and pit formation caused by resorption of mature osteoclastic bone. However, FAEE suppressed osteoclastogenesis, as well as the formation of F-actin rings and pits in RAW 264.7 cells without inhibiting cell viability. Thus, we further investigated the underlying mechanisms of how FAEE affects the in differentiation and functions of osteoclasts.

Mounting evidence indicates that during osteoclastogenesis, NFATc1 is a ‘master executor’ of RANKL-induced osteoclast differentiation/activation (Takayanagi 2007a; Sakai et al. 2016). Stimulation of RAW 264.7 cells by RANKL increases NFATc1 expression (Wang et al. 2018). NFATc1 is also a downstream nuclear transcription factor that plays a vital part in regulating expression of the osteoclast-specific genes, *TRAP, cathepsin K, MMP-9, c-Src, OSCAR* and *ATP6v0d2* (Takayanagi 2007a, 2007b; Chen et al. 2014), which are related to regulation of the differentiation, fusion and activation of osteoclasts. We showed that FAEE suppressed the RANKL-mediated stimulation of expression of NFATc1 mRNA and protein, and concurrently inhibited mRNA expression of NFATc1-mediated osteoclastogenic genes.

Osteoclastogenesis is a complex process involving the proliferation, fusion, maturation and migration of cells (Boyle et al. 2003). When RANKL binds to RANK, it immediatedly recruits TRAF-6, which activates the downstream signalling pathways regulating osteoclast differentiation/formation (Lacey et al. 1998), such as MAPKs (Boyce et al. 1999). The MAPK signalling pathway is a vital signal transmitter from the cell surface to the nucleus, it controls cell differentiation, cell growth, the inflammatory responses and environmental stresses (Shaul and Seger 2007; Boutros et al. 2008). The MAPK signalling pathway plays an important part in osteoclast differentiation (Miyazaki et al. 2000); it regulates important downstream regulatory factors, such as c-Fos and NFATc1, during osteoclast formation and, ultimately, stimulates osteoclast differentiation (Liao et al. 2021). The MAPK signalling pathway includes ERK, JNK, and p38 pathways (Yang et al. 2021). In addition, the phosphorylation of MAPKs (ERK, JNK and p38) is conducive to RANKL-stimulated osteoclastogenesis and bone resorption. Moreover, several studies have pointed out that specific inhibitors of ERK, JNK and p38 can abrogate RANKL-induced osteoclastogenesis (Ang et al. 2011). ERK has a key role in osteoclast survival (Miyazaki et al. 2000); inhibition of ERK phosphorylation reduces the polarity and survival of mature osteoclasts (Nakamura et al. 2003). Several studies have demonstrated that JNK and p38 promote osteoclast differentiation (Kim et al. 2020). JNK-deficient rats cannot achieve osteoclast differentiation (David et al. 2002); one study showed that severe osteoporosis is caused by a lack of normal bone resorption in mice lacking Mapk8 (Ikeda et al. 2004). Moreover, p38 affects the phase of osteoclast differentiation and is associated with expression of cathepsin K (Matsumoto et al. 2000; 2004), and p38 deficiency has been shown to results in an osteoporotic phenotype in 6-month-old mice (Cong et al. 2017). We demonstrated that FAEE inhibited the MAPK signalling pathway by suppressing the phosphorylation of ERK, JNK and p38.

OVX Sprague-Dawley rats are used widely as experimental models of postmenopausal osteoporosis that can mirror bone loss in humans caused by oestrogen deficiency (Jee and Yao 2001; Lelovas et al. 2008). Thus, an OVX-induced rat model was utilised in the present study to explore the effects of FAEE treatment upon osteoporosis. BMD and BMC are important indicators of osteoporosis that evaluate the rigidity and quality of bone (Rachoń et al. 2007). Measurement of these two indicators using DEXA is the ‘gold standard’ for assessment of individuals at risk for osteoporosis, because it is the best predictor of fracture risk in people who have never broken a bone (Cummings et al. 2002; Biver et al. 2012). Therefore, we measured the BMD and BMC of the whole skeleton and femur using DEXA. Consistent with previous studies, we showed significant reduction in the BMD and BMC of the whole skeleton and femur in OVX rats after 12 weeks compared with those in sham-operated rats (Ikeda et al. 2012). However, our results suggest that FAEE administration prevented systemic and femoral reduction in these indicators. Studies have shown that OVX rats experience a reduction in serum oestrogen levels (Kang et al. 2017). In accordance with other studies, we discovered that the serum oestrogen level in model group rats was significantly lower than that in rats in the sham-operated group, and that FAEE treatment led to a marked increase in the serum oestrogen level compared with that in the model group. The effect of FAEE was also observed with E_2,_ a positive control, which is subordinated to the research compound for exhibiting oestrogenic activity on bone modelling and remodelling (Zhang et al. 2012; Li et al. 2016). Moreover, Ca^+2^ is a phenotypic marker of bone formation (Wahba and Al-Zahrany 2013). Consistent with other studies, we demonstrated that ovariectomy led to a significant reduction in the Ca^+2^ level in serum (Wang et al. 2017). Interestingly, FAEE could help to maintain the Ca^+2^ level. ALP is typical marker of bone turnover (Kuo and Chen 2017). Studies have shown increased serum levels of ALP in OVX rats (Park et al. 2017); in our study, FAEE treatment tended to reduce ALP levels in serum. Those data indicate that FAEE can inhibit the bone turnover in OVX rats.

Inflammation is a potential risk factor for osteoporosis. The proinflammatory cytokines IL-1β plays an important part in bone resorption (Al-Daghri et al. 2017). *In vivo* and *in vitro* studies have suggested that increases in levels of proinflammatory cytokines promote bone resorption by several mechanisms (Orsal et al. 2013), such as suppressing osteoblast survival, enhancing the activation, differentiation and survival of osteoclasts, and increasing RANKL expression (Pacifici 1996; Manolagas 2000; Clowes et al. 2005). We showed that FAEE decreased serum levels of IL-1β. Grassi and co-workers demonstrated that the IL-1β serum level was increased significantly in OVX-induced rats compared with that in sham-operated rats (Grassi et al. 2007). In addition, loss of trabecular bone loss in OVX rats is due to oestrogen deficiency, and such bone loss is a major cause of fracture (Kalu 1991). Our results are consistent with findings showing that ovariectomy results in a reduced trabecular bone area in the femurs of OVX rats compared with that in sham-operated rats (Xu et al. 2019). However, in this respect, administrations of FAEE or E_2_ for 12 weeks inhibited the loss of trabecular bone area. Therefore, our data demonstrated that FAEE protects bone quality.

Collectively, we revealed the anti-osteoporosis effects of FAEE *in vitro* and *in vivo*. We demonstrated that FAEE can inhibit osteoclast differentiation and bone resorption in RANKL-induced RAW 264.7 cells, with no inhibition of cell viability, via regulation of the MAPK signalling pathway. Our *in vivo* study suggests that daily oral administration of FAEE for 12 weeks can significantly enhance BMD, serum levels of Ca and oestrogen, improve the organ coefficient of the uterus and femur, as well as reduce the level of the proinflammatory cytokine IL-1β. FAEE treatment also helped to improve the bone structure of rats with osteoporosis, increases their histomorphology, and had an important role in osteoporosis treatment. These data suggest that FAEE could be a potential therapeutic candidate for osteoporosis.

## Conclusion

In conclusion, FAEE can inhibit the RANKL-induced osteoclastogenesis *in vitro* and bone loss *in vivo* and its mechanism may be via downregulation of the MAPK signalling pathway. These findings lay a foundation for further study of FAEE.
